# Supplementary material for: Nearby armed conflict affects girls’ education in Africa
Source: PLoS One. 2025 Jan 15;20(1):e0314106. doi: 10.1371/journal.pone.0314106 (PMC11734919; doi:10.1371/journal.pone.0314106)
Supplement: S1 Table — Summary statistics on the respondents (male and female, age 15-18) in each survey year, including the number of clusters they reside in, and the proportion of these respondents that were exposed to any conflict during the ages of 6-12. (PDF) [file pone.0314106.s001.pdf]

Country	Survey year	Number aged 15-18	Number of clusters	Proportion experiencing conflict
Angola	2015	3878	616	0.1609
Benin	2001	1287	237	0.0000
Benin	2017	4012	538	0.0000
Burkina Faso	2003	2791	393	0.0000
Burundi	2016	4755	551	0.5272
Cameroon	2004	2782	449	0.1384
Cameroon	2018	3881	427	0.0278
Congo	2007	2068	288	0.5484
Egypt	2000	86	79	0.6744
Egypt	2003	124	101	0.6855
Egypt	2005	316	249	0.6424
Egypt	2008	216	168	0.4352
Gambia	2019	3024	280	0.3674
Ghana	2003	1714	399	0.0344
Ghana	2008	1556	388	0.0096
Guinea	2005	1867	289	0.0911
Guinea	2018	2832	393	0.2285
Kenya	2003	1769	373	0.3132
Kenya	2008	2048	373	0.1865
Kenya	2014	6737	1469	0.3853
Kenya	2022	6741	1534	0.3422
Lesotho	2004	1917	375	0.1017
Lesotho	2009	2128	393	0.0804
Liberia	2007	1543	273	0.6630
Liberia	2019	2043	314	0.0000
Madagascar	2008	4813	583	0.1700
Madagascar	2021	4417	646	0.0380
Mali	2001	1997	380	0.3480
Mali	2006	3186	404	0.0477
Mali	2018	2433	342	0.3679
Mauritania	2020	3397	1067	0.2170
Morocco	2003	2678	474	0.0000
Namibia	2000	1221	242	0.0049
Namibia	2006	2530	458	0.0933
Nigeria	2003	1580	347	0.1987
Nigeria	2008	7377	880	0.2975
Nigeria	2018	9114	1358	0.4404
Rwanda	2005	2775	455	0.8829
Rwanda	2019	3811	499	0.2726
Senegal	2005	3208	366	0.1269
Sierra Leone	2008	1348	337	0.9362
Sierra Leone	2019	3654	542	0.0000
South Africa	2016	1628	555	0.0000
Swaziland	2006	1976	261	0.0000
Tanzania	2003	2349	341	0.0434
Tanzania	2007	3290	463	0.3024
Tanzania	2015	3079	597	0.0003
Uganda	2000	1117	247	0.1513
Uganda	2006	1742	331	0.4730
Uganda	2011	3214	462	0.4116
Uganda	2016	4251	673	0.2129
Zambia	2007	2189	314	0.0027
Zambia	2013	5506	714	0.0005
Zambia	2018	4694	535	0.0000
Zimbabwe	1999	856	200	0.0666
Zimbabwe	2005	3189	393	0.2098
Zimbabwe	2015	3264	398	0.2102

S1 Table. Countries and survey years of the Demographic Health Surveys included in the main results. Summary statistics on the respondents (male and female, age 15-18) in each survey year, including the number of clusters they reside in, and the proportion of these respondents that were exposed to any conflict during the ages of 6-12.
