# Supplementary material for: Nearby armed conflict affects girls’ education in Africa
Source: PLoS One. 2025 Jan 15;20(1):e0314106. doi: 10.1371/journal.pone.0314106 (PMC11734919; doi:10.1371/journal.pone.0314106)
Supplement: S2 Table — Columns (1)-(2) do not include any constraints on the length of stay in current residence. The sample thus includes both non-migrants and migrants. Columns (3)-(4) display the main results in Fig 2A. These incorporate the residence condition (not moving since age 6). Columns (5)-(6) includes only individuals who had moved since age 6. All models include cluster, country-birth year, and country-birth month fixed effects. Standard errors are clustered at a DHS cluster level. *p<0.1; **p<0.05; ***p<0.01. (PDF) [file pone.0314106.s002.pdf]

	Years of schooling					
	No condition		Stayed since age 6		Moved since age 6	
	(1) Female	(2) Male	(3) Female	(4) Male	(5) Female	(6) Male
Conflict 0-25km	−0.1840***	0.0851	−0.3751***	0.1679	−0.1183	−0.0014
	(0.0679)	(0.1470)	(0.0989)	(0.2333)	(0.1974)	(0.4668)
Wealth quintile 2	0.3246***	0.4284***	0.3858***	0.5281***	0.3092***	0.5761*
	(0.0384)	(0.0919)	(0.0627)	(0.1306)	(0.1133)	(0.3137)
Wealth quintile 3	0.7786***	0.9933***	0.8092***	1.1024***	0.7845***	0.9338***
	(0.0435)	(0.0975)	(0.0735)	(0.1396)	(0.1314)	(0.3317)
Wealth quintile 4	1.3682***	1.5725***	1.4359***	1.8259***	1.4892***	1.7252***
	(0.0520)	(0.1116)	(0.0879)	(0.1708)	(0.1669)	(0.3262)
Wealth quintile 5	2.0394***	2.1607***	2.1396***	2.3853***	2.2790***	2.1222***
	(0.0612)	(0.1246)	(0.1041)	(0.2129)	(0.2109)	(0.3442)
Female head of HH	0.1603***	0.1126**	0.0982**	0.1223	0.2961***	0.2333*
	(0.0259)	(0.0553)	(0.0458)	(0.0835)	(0.0703)	(0.1331)
Household size	0.0064	−0.0091	0.0168**	−0.0005	−0.0107	−0.0208
	(0.0041)	(0.0080)	(0.0079)	(0.0133)	(0.0110)	(0.0194)
Head of HH age	0.0101***	−0.0005	0.0084***	−0.0065**	0.0084***	0.0029
	(0.0008)	(0.0018)	(0.0014)	(0.0027)	(0.0023)	(0.0041)
Mother in HH	0.5902***	0.2517***	0.3642***	0.1606**	0.8495***	0.5803***
	(0.0231)	(0.0419)	(0.0392)	(0.0681)	(0.0699)	(0.1388)
Nightlight intensity (age 6)	−0.0058	−0.0195	0.0017	−0.0342	−0.0288*	0.0093
	(0.0070)	(0.0164)	(0.0116)	(0.0347)	(0.0166)	(0.0298)
Rainfall (age 6)	0.0015*	0.0037**	0.0024	0.0076***	0.0064**	0.0043
	(0.0008)	(0.0016)	(0.0015)	(0.0027)	(0.0026)	(0.0044)
Min Temperature (age 6)	−0.0246	−0.3151	−0.2844	−0.3351	−0.1062	0.0848
	(0.1086)	(0.2216)	(0.1773)	(0.2851)	(0.3108)	(0.5125)
Max Temperature (age 6)	0.1203	0.1734	0.4256***	0.1516	−0.0441	−0.3253
	(0.0972)	(0.1923)	(0.1506)	(0.2405)	(0.2345)	(0.4653)
Observations	177,714	74,804	73,731	35,334	31,336	11,150
R^2	0.6849	0.7413	0.7355	0.7889	0.8088	0.8945
Adjusted R^2	0.5967	0.5770	0.6218	0.6079	0.6288	0.6805

S2 Table. Robustness checks for the overall effect of conflict exposure on years of schooling for females and males using different regression samples. Columns (1)-(2) do not include any constraints on the length of stay in current residence. The sample thus includes both non-migrants and migrants. Columns (3)-(4) display the main results in Fig 2A. These incorporate the residence condition (not moving since age 6). Columns (5)-(6) includes only individuals who had moved since age 6. All models include cluster, country-birth year, and country-birth month fixed effects. Standard errors are clustered at a DHS cluster level. *p<0.1; **p<0.05; ***p<0.01.
