# Supplementary material for: Nearby armed conflict affects girls’ education in Africa
Source: PLoS One. 2025 Jan 15;20(1):e0314106. doi: 10.1371/journal.pone.0314106 (PMC11734919; doi:10.1371/journal.pone.0314106)
Supplement: S3 Table — Column (1) uses a 50 km radius for conflict exposure, instead of 25 km. Column (2) removes household characteristics. Since these characteristics, taken from survey data, are measured at the time of the survey, they may have changed since the treatment (school-age conflict exposure) or may have been affected by the treatment. Column (3) removes sample weights. Sample weights are necessary to ensure that estimates are representative of all the countries in the sample, but may be sensitive to the population estimates used. Column (4) only includes observations that did not have conflict in the survey year and the year before the survey, to exclude the possibility that effects are due to more recent conflict. All models include cluster, country-birth year, and country-birth month fixed effects and use the same female sample as the main results (S2 Table, column (3)). Standard errors are clustered at a DHS cluster level. *p<0.1; **p<0.05; ***p<0.01. (PDF) [file pone.0314106.s003.pdf]

	Years of schooling			
	Conflict 50km	No covariates	No sample weights	Conflict termination
	(1)	(2)	(3)	(4)
Conflict 0-50km	−0.3886***			
	(0.1144)			
Conflict 0-25km		−0.3981***	−0.2055**	−0.3790***
		(0.1079)	(0.0817)	(0.1058)
Wealth quintile 2	0.3863***		0.3808***	0.3596***
	(0.0626)		(0.0360)	(0.0640)
Wealth quintile 3	0.8126***		0.8242***	0.7682***
	(0.0732)		(0.0396)	(0.0761)
Wealth quintile 4	1.4372***		1.3514***	1.3725***
	(0.0876)		(0.0459)	(0.0914)
Wealth quintile 5	2.1438***		2.1175***	2.0856***
	(0.1041)		(0.0578)	(0.1066)
Female head of HH	0.0985**		0.0798***	0.0902*
	(0.0457)		(0.0253)	(0.0480)
Household size	0.0169**		0.0065*	0.0147*
	(0.0079)		(0.0039)	(0.0084)
Head of HH age	0.0083***		0.0070***	0.0088***
	(0.0014)		(0.0008)	(0.0014)
Mother in HH	0.3639***		0.4376***	0.3694***
	(0.0392)		(0.0253)	(0.0401)
Nightlight intensity (age 6)	0.0018	0.0031	−0.0111	−0.0066
	(0.0116)	(0.0120)	(0.0091)	(0.0129)
Rainfall (age 6)	0.0024	0.0024	0.0011	0.0033**
	(0.0015)	(0.0015)	(0.0009)	(0.0016)
Min Temperature (age 6)	−0.2820	−0.2751	−0.1116	−0.2769
	(0.1772)	(0.1804)	(0.1073)	(0.1879)
Max Temperature (age 6)	0.4189***	0.4460***	0.1783*	0.4126**
	(0.1513)	(0.1526)	(0.0937)	(0.1651)
Observations	73,731	76,828	73,731	66,068
R^2	0.7356	0.7298	0.7133	0.7384
Adjusted R^2	0.6218	0.6117	0.5900	0.6235

S3 Table. Sensitivity of the overall effect of conflict exposure on female years of schooling to modeling assumptions. Column (1) uses a 50 km radius for conflict exposure, instead of 25 km. Column (2) removes household characteristics. Since these characteristics, taken from survey data, are measured at the time of the survey, they may have changed since the treatment (school-age conflict exposure) or may have been affected by the treatment. Column (3) removes sample weights. Sample weights are necessary to ensure that estimates are representative of all the countries in the sample, but may be sensitive to the population estimates used. Column (4) only includes observations that did not have conflict in the survey year and the year before the survey, to exclude the possibility that effects are due to more recent conflict. All models include cluster, country-birth year, and country-birth month fixed effects and use the same female sample as the main results (S2 Table, column (3)). Standard errors are clustered at a DHS cluster level. $^{*}p<0.1$; $^{**}p<0.05$; $^{***}p<0.01$.
