# Supplementary material for: Nearby armed conflict affects girls’ education in Africa
Source: PLoS One. 2025 Jan 15;20(1):e0314106. doi: 10.1371/journal.pone.0314106 (PMC11734919; doi:10.1371/journal.pone.0314106)
Supplement: S4 Table — Regression uses a binary variable for whether or not the individual has completed at least a primary education, as is sometimes used in the literature, instead of Years of schooling. Coefficient estimates are from logistic regression on the female sample in the main results (S2 Table, column (3)). Standard errors are clustered at a DHS cluster level. *p<0.1; **p<0.05; ***p<0.01. (PDF) [file pone.0314106.s004.pdf]

Dependent Variable:	At least primary
Model:	(1)
Variables	
Conflict 0-25km	-0.4193*
	(0.2437)
Wealth quintile 2	0.5358***
	(0.0999)
Wealth quintile 3	1.027***
	(0.1098)
Wealth quintile 4	1.698***
	(0.1233)
Wealth quintile 5	2.512***
	(0.1600)
Female head of HH	0.0402
	(0.0757)
Household size	0.0100
	(0.0118)
Head of HH age	0.0071***
	(0.0022)
Mother in HH	0.4097***
	(0.0621)
Nightlight intensity (age 6)	0.0337
	(0.0214)
Rainfall (age 6)	0.0017
	(0.0028)
Min Temperature (age 6)	-0.7425**
	(0.2910)
Max Temperature (age 6)	0.7403***
	(0.2634)
Fixed-effects	
DHS cluster	Yes
Country-Birth year	Yes
Country-Birth month	Yes
Observations	73,768

S4 Table. Alternative outcome variable for educational attainment. Regression uses a binary variable for whether or not the individual has completed at least a primary education, as is sometimes used in the literature, instead of Years of schooling. Coefficient estimates are from logistic regression on the female sample in the main results (S2 Table, column (3)). Standard errors are clustered at a DHS cluster level. $^{*}p<0.1$; $^{**}p<0.05$; $^{***}p<0.01$.
