# Supplementary material for: Nearby armed conflict affects girls’ education in Africa
Source: PLoS One. 2025 Jan 15;20(1):e0314106. doi: 10.1371/journal.pone.0314106 (PMC11734919; doi:10.1371/journal.pone.0314106)
Supplement: S5 Table — Regressions use different outcome variables for different potential mechanisms: column (1) uses a binary variable taking the value 1 if the individual has never been married. Column (2) uses a binary variable taking the value 1 if the individual’s mother is still alive, and column (3) for the father. Columns (2) and (3) remove the controls for female head of household and whether or not the mother is in the household, since these are highly correlated with the outcome variables. Coefficient estimates are from logistic regression on the female sample in the main results (S2 Table, column (3)). Standard errors are clustered at a DHS cluster level. *p<0.1; **p<0.05; ***p<0.01. (PDF) [file pone.0314106.s005.pdf]

| Dependent Variables: Model: | Never Married (1) | Mother Alive (2) | Father Alive (3) |
|---|---|---|---|
| Variables | | | |
| Conflict 0-25km | 0.3183 | 0.6239 | -0.0146 |
| | (0.4075) | (0.4490) | (0.2619) |
| Wealth quintile 2 | 0.0367 | 0.2534 | 0.2019* |
| | (0.1677) | (0.1849) | (0.1153) |
| Wealth quintile 3 | 0.2505 | 0.3825** | 0.5332*** |
| | (0.1926) | (0.1950) | (0.1295) |
| Wealth quintile 4 | 0.0794 | 0.1026 | 0.8009*** |
| | (0.2549) | (0.2153) | (0.1511) |
| Wealth quintile 5 | 0.5987* | 0.5226* | 0.9180*** |
| | (0.3391) | (0.2828) | (0.2041) |
| Female head of HH | 0.9982*** | | |
| | (0.1518) | | |
| Household size | 0.1724*** | 0.0950*** | 0.1836*** |
| | (0.0178) | (0.0196) | (0.0168) |
| Head of HH age | 0.0791*** | -0.0121*** | 0.0119*** |
| | (0.0045) | (0.0040) | (0.0031) |
| Mother in HH | 2.907*** | | |
| | (0.1524) | | |
| Nightlight intensity (age 6) | 0.0717 | -0.0685 | 0.0149 |
| | (0.0563) | (0.0461) | (0.0320) |
| Rainfall (age 6) | -0.0077* | 0.0028 | 0.0023 |
| | (0.0045) | (0.0053) | (0.0034) |
| Min Temperature (age 6) | -0.7518 | 0.9027 | 0.2944 |
| | (0.5389) | (0.5596) | (0.3805) |
| Max Temperature (age 6) | 0.1723 | -0.3577 | -0.1955 |
| | (0.4977) | (0.4700) | (0.3112) |
| Fixed-effects | | | |
| DHS cluster | Yes | Yes | Yes |
| Country-Birth year | Yes | Yes | Yes |
| Country-Birth month | Yes | Yes | Yes |
| Observations | 73,768 | 48,110 | 47,794 |

**S5 Table. Mechanisms for the effect of conflict exposure on female years of schooling.** Regressions use different outcome variables for different potential mechanisms: column (1) uses a binary variable taking the value 1 if the individual has never been married. Column (2) uses a binary variable taking the value 1 if the individual's mother is still alive, and column (3) for the father. Columns (2) and (3) remove the controls for female head of household and whether or not the mother is in the household, since these are highly correlated with the outcome variables. Coefficient estimates are from logistic regression on the female sample in the main results (S2 Table, column (3)). Standard errors are clustered at a DHS cluster level. *p<0.1; **p<0.05; ***p<0.01.
