# Supplementary material for: Nearby armed conflict affects girls’ education in Africa
Source: PLoS One. 2025 Jan 15;20(1):e0314106. doi: 10.1371/journal.pone.0314106 (PMC11734919; doi:10.1371/journal.pone.0314106)
Supplement: S6 Table — The outcome variables in S5 Table potentially affect whether or not an individual is still cohabitating with a member of the older generation. As explained in Materials and Methods, the length of residence condition, used in the main sample (and in S5 Table), requires that individuals still be cohabitating with the older generation. Regressions in this table remove the length of residence condition (corresponding to overall effects in columns (1)-(2) of S2 Table), so individuals who moved since age 6 are included in the sample. The effect of conflict exposure remains insignificant, and do not change the conclusion that there is no evidence to suggest that conflict exposure increases the likelihood of child marriages or parental deaths. Coefficient estimates are from logistic regression. Standard errors are clustered at a DHS cluster level. *p<0.1; **p<0.05; ***p<0.01. (PDF) [file pone.0314106.s006.pdf]

| Dependent Variables:<br>Model: | Never Married<br>(1) | Mother Alive<br>(2) | Father Alive<br>(3) |
|---|---|---|---|
| Variables | | | |
| Conflict 0-25km | -0.0850 | 0.1496 | -0.0556 |
| | (0.1983) | (0.2071) | (0.1417) |
| Wealth quintile 2 | 0.0293 | -0.0272 | 0.2576*** |
| | (0.0815) | (0.1032) | (0.0683) |
| Wealth quintile 3 | 0.2694*** | -0.0623 | 0.4639*** |
| | (0.0939) | (0.1058) | (0.0783) |
| Wealth quintile 4 | 0.4858*** | -0.0689 | 0.6733*** |
| | (0.1176) | (0.1176) | (0.0844) |
| Wealth quintile 5 | 1.167*** | -0.1396 | 0.9138*** |
| | (0.1476) | (0.1374) | (0.1029) |
| Female head of HH | 0.9091*** | | |
| | (0.0673) | | |
| Household size | 0.1056*** | 0.0530*** | 0.1017*** |
| | (0.0077) | (0.0089) | (0.0070) |
| Head of HH age | 0.0594*** | -0.0043** | 0.0115*** |
| | (0.0018) | (0.0020) | (0.0015) |
| Mother in HH | 3.013*** | | |
| | (0.0724) | | |
| Nightlight intensity (age 6) | 0.0111 | -0.0127 | -0.0005 |
| | (0.0197) | (0.0186) | (0.0144) |
| Rainfall (age 6) | 0.0010 | 0.0012 | -0.0008 |
| | (0.0022) | (0.0022) | (0.0016) |
| Min Temperature (age 6) | -0.3274 | 0.4697* | 0.1968 |
| | (0.2696) | (0.2849) | (0.2148) |
| Max Temperature (age 6) | 0.1652 | 0.2331 | -0.0679 |
| | (0.2412) | (0.2502) | (0.1791) |
| Fixed-effects | | | |
| DHS cluster | Yes | Yes | Yes |
| Country-Birth year | Yes | Yes | Yes |
| Country-Birth month | Yes | Yes | Yes |
| Observations | 81,633 | 36,483 | 61,071 |

**S6 Table. Sensitivity of the results in S5 Table to the length of residence condition.** The outcome variables in S5 Table potentially affect whether or not an individual is still cohabitating with a member of the older generation. As explained in *Materials and Methods*, the length of residence condition, used in the main sample (and in S5 Table), requires that individuals still be cohabitating with the older generation. Regressions in this table remove the length of residence condition (corresponding to overall effects in columns (1)-(2) of S2 Table), so individuals who moved since age 6 are included in the sample. The effect of conflict exposure remains insignificant, and do not change the conclusion that there is no evidence to suggest that conflict exposure increases the likelihood of child marriages or parental deaths. Coefficient estimates are from logistic regression. Standard errors are clustered at a DHS cluster level. $^{*}$p<0.1; $^{**}$p<0.05; $^{***}$p<0.01.
