# Supplementary material for: Nearby armed conflict affects girls’ education in Africa
Source: PLoS One. 2025 Jan 15;20(1):e0314106. doi: 10.1371/journal.pone.0314106 (PMC11734919; doi:10.1371/journal.pone.0314106)
Supplement: S7 Table — The outcome variable in the regression is based on responses to a question on current school attendance in the household portion of the DHS. The regression outcome variable is coded 1 if the respondent was currently attending or attended school at some time in the current school year. If it were the case that schooling is delayed due to conflict, so losses in schooling might be reversed by adulthood, we would expect positive effects on school attendance at age 15-18. Here we do not find significant effects. Coefficient estimates are from logistic regression on the female sample in the main results (S2 Table, column (3)). Standard errors are clustered at a DHS cluster level. *p<0.1; **p<0.05; ***p<0.01. (PDF) [file pone.0314106.s007.pdf]

Dependent Variable:	Attending School
Model:	(1)
Variables	
Conflict 0-25km	0.2849
	(0.1780)
Wealth quintile 2	0.5491***
	(0.1021)
Wealth quintile 3	0.9318***
	(0.1122)
Wealth quintile 4	1.519***
	(0.1341)
Wealth quintile 5	1.857***
	(0.1622)
Female head of HH	0.0149
	(0.0751)
Household size	0.0382***
	(0.0095)
Head of HH age	0.0115***
	(0.0023)
Mother in HH	0.8159***
	(0.0693)
Nightlight intensity (age 6)	-0.0128
	(0.0209)
Rainfall (age 6)	-0.0043
	(0.0029)
Min Temperature (age 6)	-0.5437*
	(0.3170)
Max Temperature (age 6)	0.0234
	(0.2792)
Fixed-effects	
DHS cluster	Yes
Country-Birth year	Yes
Country-Birth month	Yes
Observations	173,680

S7 Table. Effect of conflict exposure on school attendance at age 15-18. The outcome variable in the regression is based on responses to a question on current school attendance in the household portion of the DHS. The regression outcome variable is coded 1 if the respondent was currently attending or attended school at some time in the current school year. If it were the case that schooling is delayed due to conflict, so losses in schooling might be reversed by adulthood, we would expect positive effects on school attendance at age 15-18. Here we do not find significant effects. Coefficient estimates are from logistic regression on the female sample in the main results (S2 Table, column (3)). Standard errors are clustered at a DHS cluster level. *p<0.1; **p<0.05; ***p<0.01.
