# Supplementary material for: Nearby armed conflict affects girls’ education in Africa
Source: PLoS One. 2025 Jan 15;20(1):e0314106. doi: 10.1371/journal.pone.0314106 (PMC11734919; doi:10.1371/journal.pone.0314106)
Supplement: S8 Table — Column (1) contains the subset of the female sample, used in the main results, that was born in 1983-1996. Column (2) contains the subset that was born in 1997-2007. The rates of conflict exposure in the earlier and later birth cohorts are similar at 21.2% and 21.6%. All models include cluster, country-birth year, and country-birth month fixed effects. Standard errors are clustered at a DHS cluster level. *p<0.1; **p<0.05; ***p<0.01. (PDF) [file pone.0314106.s008.pdf]

	Years of schooling	
	Cohorts 1983-1996	Cohorts 1997-2007
	(1)	(2)
Conflict 0-25km	-0.3540^{**}	-0.3918^{***}
	(0.1471)	(0.1382)
Wealth quintile 2	0.3077^{***}	0.4854^{***}
	(0.0939)	(0.0816)
Wealth quintile 3	0.7231^{***}	0.9342^{***}
	(0.1110)	(0.0936)
Wealth quintile 4	1.2951^{***}	1.6001^{***}
	(0.1297)	(0.1137)
Wealth quintile 5	2.1001^{***}	2.2258^{***}
	(0.1513)	(0.1422)
Female head of HH	0.0678	0.1230^{**}
	(0.0739)	(0.0519)
Household size	0.0052	0.0285^{***}
	(0.0125)	(0.0094)
Head of HH age	0.0081^{***}	0.0088^{***}
	(0.0021)	(0.0020)
Mother in HH	0.3758^{***}	0.3511^{***}
	(0.0628)	(0.0502)
Nightlight intensity (age 6)	−0.0137	0.0117
	(0.0175)	(0.0154)
Rainfall (age 6)	0.0013	0.0021
	(0.0030)	(0.0015)
Min Temperature (age 6)	−0.3551	-0.5433^{**}
	(0.2643)	(0.2284)
Max Temperature (age 6)	0.1679	0.8042^{***}
	(0.2152)	(0.2078)
Observations	34,609	39,122
R^2	0.7420	0.7266
Adjusted R^2	0.6260	0.6044

S8 Table. Effect of conflict exposure on female years of schooling for different birth cohorts. Column (1) contains the subset of the female sample, used in the main results, that was born in 1983-1996. Column (2) contains the subset that was born in 1997-2007. The rates of conflict exposure in the earlier and later birth cohorts are similar at 21.2% and 21.6%. All models include cluster, country-birth year, and country-birth month fixed effects. Standard errors are clustered at a DHS cluster level. *p<0.1; **p<0.05; ***p<0.01.
