# Supplementary figures and images for: Nearby armed conflict affects girls’ education in Africa
Source: PLoS One. 2025 Jan 15;20(1):e0314106. doi: 10.1371/journal.pone.0314106 (PMC11734919; doi:10.1371/journal.pone.0314106)

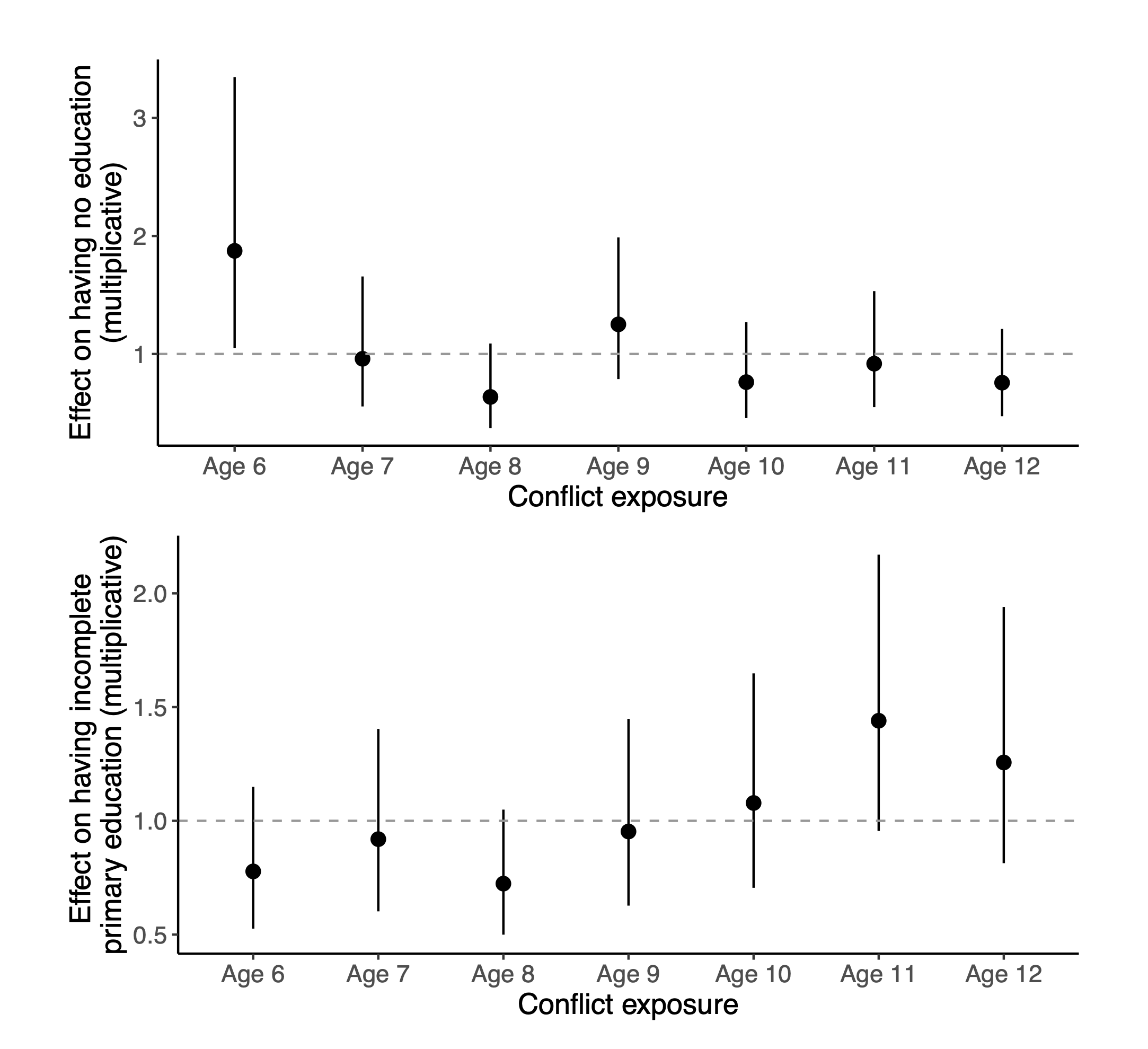

Supplement: S1 Fig — Conflict in each year is defined as the occurrence of a violent event within 25 km in each year that the respondent turned 6 to 12. Exponentiated regression coefficients, plotted on the y axis, indicate the multiplicative increase in the odds of having no education (top), and incomplete primary education (bottom). Estimation is by logistic regression and bars are 95% confidence intervals. (TIF) [file pone.0314106.s009.tif]

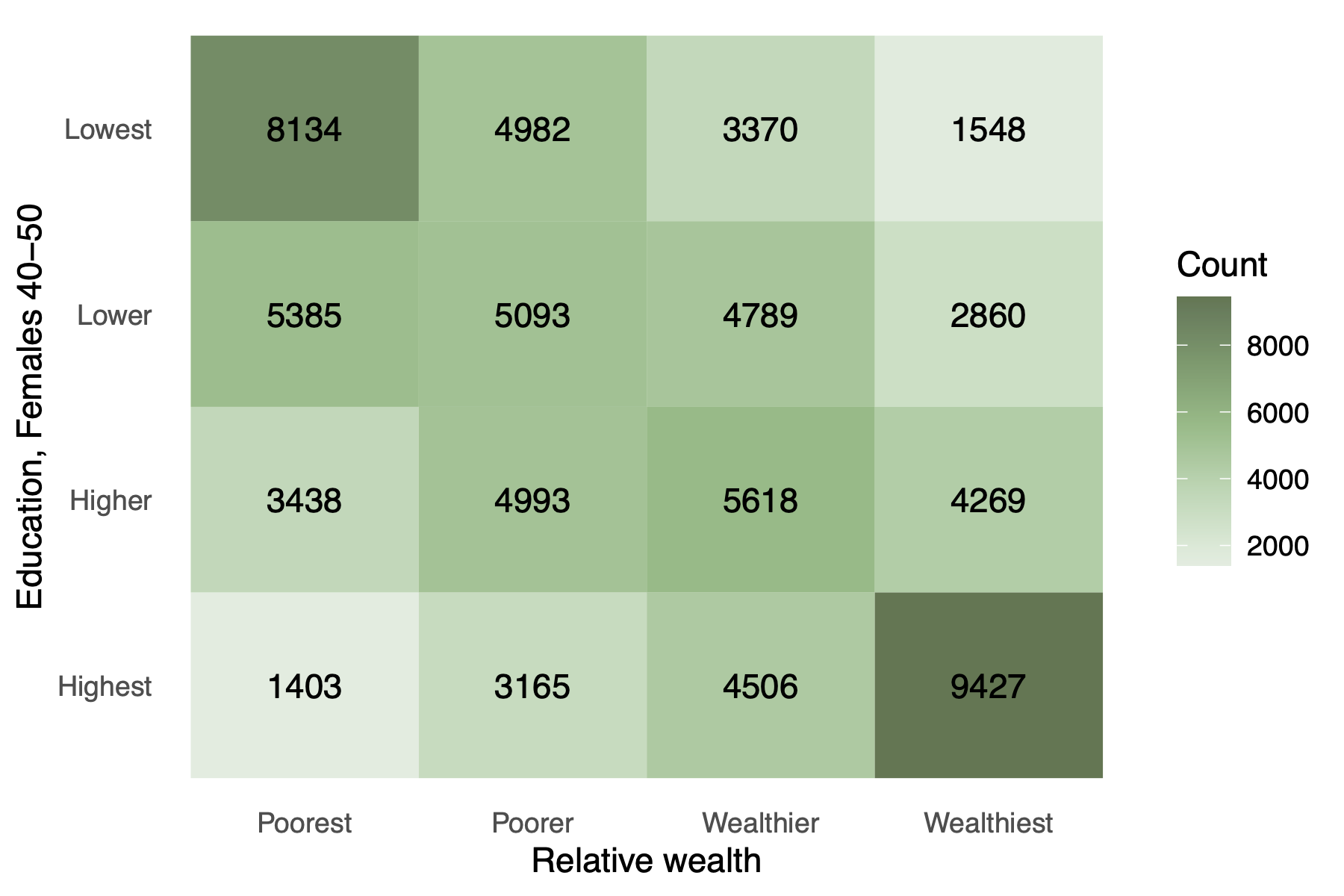

Supplement: S2 Fig — Wealth quartiles are based on cluster mean relative wealth index. Education quartiles are based on cluster mean years of schooling for females aged 40-50. (TIF) [file pone.0314106.s010.tif]

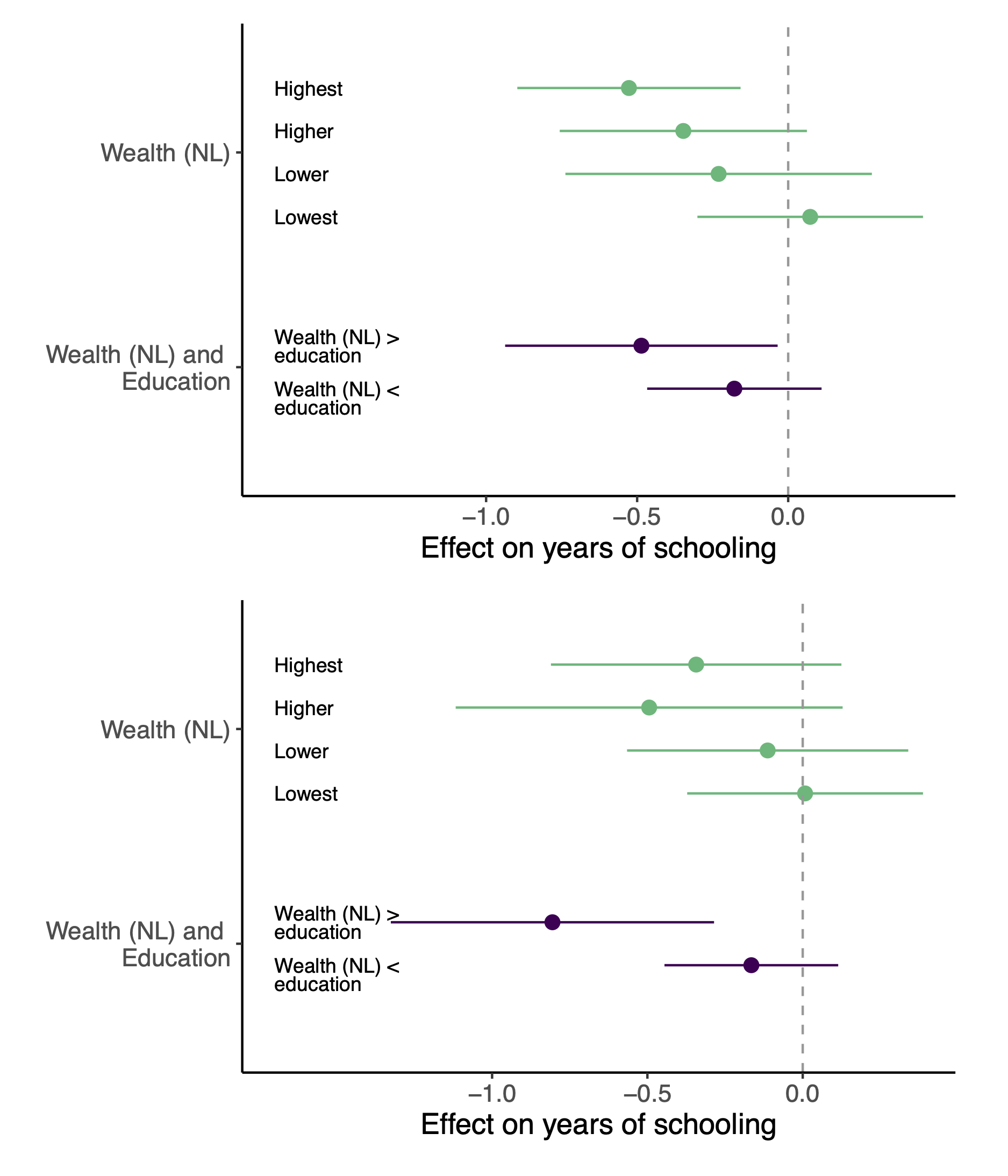

Supplement: S3 Fig — Top: wealth quartiles are based on nightlight intensity at the time of the survey, rather than DHS wealth index at the time of the survey in Fig 4. Bottom: wealth quartiles are based on nightlight intensity at age 6. Measuring wealth at age 6 instead of at the time of the survey addresses the concern than wealth may be affected by conflict occurrence and is thus endogenous. Here we see that the results are qualitatively unchanged. Bars are 95% confidence intervals. (TIF) [file pone.0314106.s011.tif]

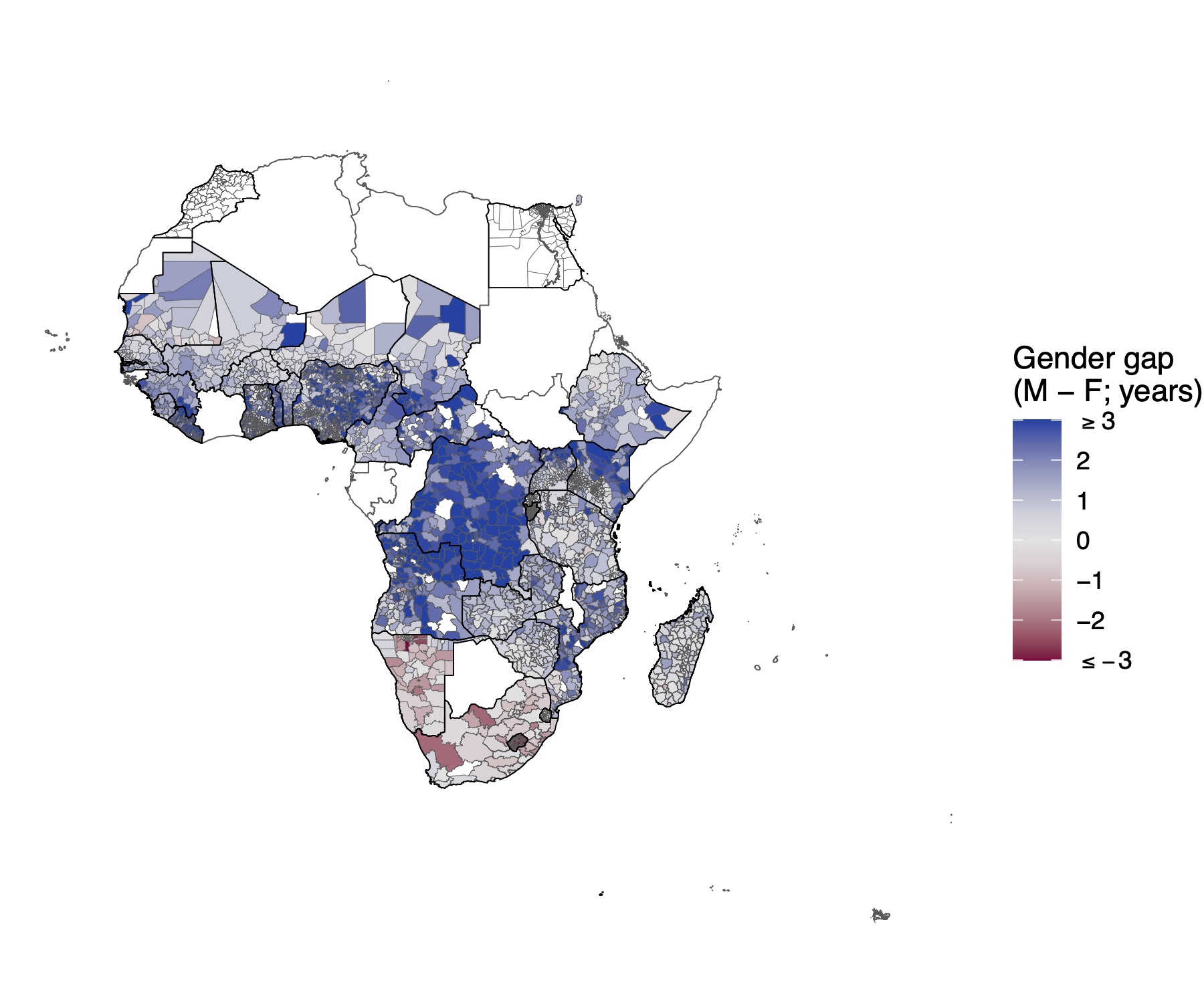

Supplement: S4 Fig — Gender gap is the difference between mean years of schooling among all males and females surveyed in each administrative level 2 region. Regions with no data are colored in white. Administrative boundaries are from [20]. (TIF) [file pone.0314106.s012.tif]
