# Supplementary material for: Nearby armed conflict affects girls’ education in Africa
Source: PLoS One. 2025 Jan 15;20(1):e0314106. doi: 10.1371/journal.pone.0314106 (PMC11734919; doi:10.1371/journal.pone.0314106)
Supplement: Attachment — Submitted filename: ResponseReviewers.pdf [file pone.0314106.s013.pdf]

Response to R1 (PLOS ONE #PONE-D-24-25168)

R1's comments are in black/Arial; point-by-point responses are in blue/Times

Overall, this Research Article is a compelling treatment of the complex relationship between the impact of conflict on female educational attainment in 28 African countries. It is rich with data, data analysis -- plus justification of data selected and systematically applied. The lone author clearly states the purpose of the research, explains his methodology with insightful details -- and remains on course to results and discussion with clear cut regression analysis. The research presented is technically sound with logical justification -- and reveals and confirms a measurable loss of educational attainment (0.4 years) for females in selected African countries having experienced conflict. Also, the Research Article is a timely and reasonable body od knowledge for developing policy to mitigate the impact of conflict on educational attainment in struggling countries.

> Thank you for your enthusiastic assessment of this manuscript, and for taking the time to review it!

R2's comments are in black/Arial; point-by-point responses are in blue/Times

Thank you for taking the time to review this paper. Below are point-by-point responses to each of your comments. You may also notice other changes, which were made in response to comments from the editor and other reviewers. I think the result is a significantly improved manuscript. Thanks again!

Comment 1:
Thank you for giving me the opportunity to review this interesting paper. The study aims to estimate the impact of local conflict events on the educational attainment of girls in Africa. The writing and presentation are commendable. However, I have one major concern and one minor comment. With some improvements, I believe this paper could be of great interest to the journal's readership.

Major
As the author is aware, the paper estimates correlation, which does not necessarily imply causation. While several robustness checks have been performed, the study fails to establish of a clear counterfactual. I understand that some may claim causality using OLS with strong assumptions, but it would be beneficial for the author to explore exogenous variation in the treatment. For example, a Regression Discontinuity Design (RDD) using distance to the conflict event as the running variable, with 25 km as the cut-off, could be considered (this is just an example). This is a significant drawback of the study. If the author decides to proceed with the current estimation strategy, they should clearly state the strategy, its limitations, and the strong assumptions made to claim causality in the abstract, introduction, and conclusion sections.

Thank you for this comment. I understand and agree with your general reservations about the use of observational data. In my response I first clarify how the assumptions, while appearing to be strong, are not implausible in this specific scenario. Next, I address your point about exploring exogenous variation in treatment. Finally, I explain changes in the text to improve the clarity of identification assumptions.

The main assumption behind the identification strategy used in this study is that there are no remaining unmeasured confounders, after controlling for variables including cluster fixed effects, country-specific time fixed effects, and time-varying factors such as nightlight intensity, which could plausibly affect both the occurrence of conflict and educational outcomes. The main threat to identification is that there are remaining confounders that affect both conflict (at ages 6-12) and educational outcomes (measured at age 15-18), that vary within the same DHS cluster over time (birth year). Note first that if these unmeasured factors vary only slowly over time, the issue is less concerning, since they might approximate time-invariant factors that cluster fixed effects account for. Summarizing more plainly, the assumption is that conflict occurs in a cluster, and some individuals, out of randomness, happen to be of school-going age when it occurs, while others are not; we hence attribute differences in educational outcomes to conflict occurrence.

Now, a time-varying confounder that threatens identification would have to act on individuals in the same cluster but in different birth years, affecting their conflict

exposure at ages 6-12 and resulting years of schooling. For example, imagine a religion that is adhered to intermittently, where every time it is adhered to, there is no conflict, and females do not attend school. In this scenario, the effect of conflict will appear to be positive, although this is not a causal relationship, since it is a result of the unobserved religion. Because of the scope of the data used in this study, such a confounding variable will need to act on a significant enough subset of the 28 countries and 25 birth cohorts to bias the results. I am not aware of a confounding variable that fulfils these criteria, but I acknowledge the possibility of its existence.

Next, I have considered looking for strategies that provide exogenous variation in the treatment like you are suggesting, but unfortunately there is not an obvious study design that provides this. Due to the nature of empirical work on conflict, this is an issue shared by other researchers working on similar topics, and as a result, strategies relying on OLS and fixed effects have been used in multiple studies. A few examples that are most closely related and use the same UCDP and DHS data sources include studies examining the impact of conflict on child deaths (Wagner et al. 2018), maternal deaths (Kotsadam and Østby 2019), and malaria risk (Yu et al. 2024). All these studies rely on OLS and fixed effects, due to difficulties in finding a source of exogenous variation in conflict exposure.

Thank you for the specific suggestion of an RDD using distance to conflict event as a running variable. This is a promising idea but runs into several difficulties in this application. First, there is not a clear idea of a discontinuity in treatment assignment at a specific distance. Fig 2B suggests that 50 km might be an appropriate threshold since the average effect is not significantly different from zero beyond this distance, however this is an average effect that may differ depending on severity of the violent event and location-specific characteristics. Next, an individual could experience multiple conflict events at varying distances, so it is not immediately obvious how the proposed running variable for distance to event should be constructed. Further, the geo-coordinates of violent events are measured with error – for roughly 30% of the violent events that we consider, the exact coordinates are not known, rather the events are known to have occurred within a 25 km radius of a known point. These issues make it difficult to accurately construct a running variable that is suitable for use in an RD design. Spatial RD designs tend to use arbitrary boundaries, e.g., Dell 2010 uses boundaries of a forced mining labor system, and Melnikov et al. 2022 uses boundaries of gang-controlled areas. One possibility here is to consider country borders, state or county boundaries, or school districts --- perhaps violent events that are within 25 km but occur on the other side of these boundaries have different effects. This idea is speculative at this point, and would require significant new analysis and data-gathering to flesh out fully. At this stage, I am reluctant to completely change the identification strategy and scope of the paper. I agree that other study designs could be explored in future research and if successful would be an important contribution to the set of strategies used in empirical conflict research.

In summary, rather than searching for a perfect identification strategy, I think a potentially imperfect strategy can still contribute useful insights. I take your point that the assumptions could be stated more prominently, and have made changes to the abstract, introduction and conclusion. In the abstract, I clarify that the paper uses a regression-based approach: "We link granular data on conflict events to georeferenced survey data on educational attainment from 28 countries in Africa, and use a regression-based approach to estimate the local effect of conflict exposure on female years of schooling."

I moved the paragraph describing the identification strategy from the Results to the Introduction, and added an explanation of the required assumptions and limitations. The paragraph now reads:

> Since many factors may contribute to associations between conflict exposure and years of schooling, we use a regression framework that accounts for location-specific factors (e.g., certain areas being less likely to experience conflict and having a higher female educational attainment), cohort effects (e.g., global events), time-varying factors such as climate and rainfall, and household characteristics such as wealth. We thus compare children in the same location that experienced conflict during their primary school years, to children who did not --- a similar strategy to other recent work that examines the effect of conflict on child mortality (Wagner et al. 2018), maternal mortality (Kotsadam and Østby 2019), and immunizations (Østby et al. 2021). To interpret the estimates as causal, we rely on the assumption that there are no remaining unmeasured confounders that affect both conflict and educational outcomes, that vary within the same location over time (birth year). We believe this assumption to be plausible but acknowledge the limitations of inferring causal effects from observational data (see *Materials and Methods*).

In the conclusion, when discussing limitations, I added the following:

> An important one is that these are observational data, which require several assumptions for a causal interpretation of estimates. In particular, if there are remaining time-varying factors within DHS clusters that are unaccounted for, that are correlated with both conflict and education, this would limit the causal interpretation of our estimates.

Comment 2:
Minor
Typically, readers expect to see the materials and methods section before the results and discussion. The author may want to restructure the paper so that it follows this sequence, 1. Introduction, 2. Materials and Methods.

Thank you for this suggestion. I agree with you that the methods section typically comes before the results, in particular in discipline-specific journals. In interdisciplinary journals (such as the Nature family journals), it is more typical (sometimes required) for the full

methods section to come at the end. While PLOS ONE does not have strict requirements as to the order of the sections, I believe presenting the results first, with a brief description of the methodology, improves readability for a more general audience. People from related fields, such as sociology or psychology for example, may be less interested in the full details of the data and estimation strategy. I have hence elected to keep the order as is, but thank the reviewer for the suggestion.

R3's comments are in black/Arial; point-by-point responses are in blue/Times

Thank you for your thoughtful and constructive review. Below are point-by-point responses to each of your comments.

You may also notice other changes, which were made in response to comments from the editor and other reviewers. Thanks again for taking the time to review this work!

Comment 1:
One of the interesting and intriguing finding of the paper is the habituation to violence. This is summarised by the following quote from page 3: "On the other hand, recurring violence, defined as events occurring in consecutive years, has a positive effect at certain ages of exposure, suggesting a habituation to conflict." It is difficult to understand how consecutive years of conflict positively affect years of education; as far as we are considering marginal effects of conflict (i.e., controlling for other factors that affect conflict), shouldn't it be necessarily negative (or at best zero)?

Thank you for raising this important point. You are correct that a positive coefficient estimate seems counterintuitive. If we look at a single coefficient, say the estimate for recurring conflict at age 8 (purple bar in Fig 3D), this suggests that compared to experiencing no conflict during ages 6-12, experiencing recurring conflict at age 8 increases female years of schooling. However, this coefficient is not meant to be interpreted on its own as the overall effect of conflict exposure. The definition of recurring conflict is that it has occurred in the previous year as well, i.e., at age 7. This conflict at age 7 may be new or recurring. Hence the more appropriate way to interpret these coefficients as an overall effect is to consider a linear combination of conflict occurrences from ages 6 to 12. In the previous example where there was conflict exposure at age 7, say the person experienced conflict at ages 7, 8, 10, 11. We would then sum the coefficients for new conflict at age 7, recurring at age 8, new at age 10, and recurring at age 11. The estimated effect is then -0.13 (CI: (-0.83, 0.56)). (Note that the confidence intervals are wide since the conflict occurrence variables are necessarily correlated.)

To further illustrate this point, the following table summarizes the estimates for two additional scenarios that illustrate the type of habituation that was highlighted in the manuscript: chronic conflict (every year from 6-12) and intermittent conflict. The point estimate for chronic conflict is close to 0, and that for intermittent conflict is -0.59.

Ages experienced	Interpretation	Point estimate	Confidence interval
6-12	Chronic conflict	0.016	(-1.11, 1.14)
6, 8, 10, 12	Intermittent conflict	-0.59	(-1.34, 0.21)
7, 8, 10, 11	Above example	-0.13	(-0.83, 0.56)

I now see how my previous presentation of this positive coefficient was confusing, and I have clarified this in the text in several places. In the abstract, the relevant text reads:

> Consecutive years of conflict, however, can have positive effects in later years, which offset earlier negative effects, suggesting a habituation to violence.

Similarly in the introduction:

> On the other hand, recurring violence, defined as events occurring in consecutive years, have positive effects in later years of exposure (that offset negative effects in earlier years), suggesting a habituation to conflict.

The main explanation is in the results section:

> Note that the positive coefficient in Figure 3D does not more generally suggest that conflict increases final educational attainment -- the definition of recurring conflict is that it has also occurred in the previous year, and the positive coefficient can be interpreted as offsetting negative effects in previous years. More generally, estimating the overall effect on educational attainment will require considering a linear combination of coefficient estimates from ages 6 to 12.

Comment 2:
p. 14: "We measure outcomes at late adolescence, age 15-18, and create separate samples for females and males." Why estimate separate regressions for females and males? Isn't the better option to run on pooled data with female/male dummy? When running separate regressions, the degrees of freedom in the two samples may differ influencing the estimated results; a pooled regression with dummy variables can avoid that. Alternatively, if there is reason to suspect that female and male years of education are systematically different, it is better to run a formal test for structural break (e.g., Chow test) to justify the estimation of separate regressions.

Thank you for pointing this out. There are several reasons for creating separate samples. First, the sampling processes for women and men are slightly different. Some surveys only interview women and do not have men's records. A subset of surveys use "ever-married samples," where men are eligible for interview only if they have ever been married or lived in a consensual union. Overall, the number of men surveyed is much smaller than women. In the regression sample, the number of female observations is 73,731 and the number of males is 35,334 (these numbers are in Table S2 Columns (3)-(4)).

Second, the confounding effects could be in opposite directions for females and males. For example, the cluster fixed effects control for location-specific, time-invariant effects. Some locations may be more likely to have both conflict and higher male years of schooling, but lower female years of schooling (or vice versa). Hence a single location fixed effect for both sexes will not do an adequate job of controlling for these effects. You suggested a model with a female/male dummy – presumably this includes a female/male indicator and the same indicator interacted with conflict occurrence. To fully take into account these potentially different confounding effects, other covariates and fixed effects would have to be interacted with the female dummy as well. (A fully

interacted model would be the most similar to running separate regressions on males and females.) Experimenting with a few versions of such pooled regressions produces similar results to what is reported in the text. For example, a pooled regression with female interacted with conflict, and female interacted with location fixed effects, produces a coefficient estimate of the female*conflict of -0.39 ($p = 0.086$); including female interacted with the other fixed effects as well produces a coefficient estimate for female*conflict of -0.57 ($p = 0.022$).

Finally, thank you for the suggestion of doing a formal test. A Chow test rejects the null hypothesis that a combined model is better ($p < .001$; F statistic = 1.34 on 16311 and 70574 df).

I summarize the above in the paper (in Materials and Methods). First, I describe the samples in more detail:

> Note that the number of individual survey records for men is smaller than that for women, since in some cases, individual surveys were only conducted for women and not men. Further, a subset of surveys use "ever-married samples," where men are eligible for interview only if they have ever been married or lived in a consensual union.

When describing the regressions, I add the following:

> Males and females are treated separately for several reasons. First, the sampling processes for women and men are slightly different (see *Educational outcomes data*). Second, confounding effects could be in opposite directions for females and males. For example, some locations may be more likely to have both conflict and higher male years of schooling, but lower female years of schooling (or vice versa). A single location fixed effect for both sexes will not do an adequate job of controlling for these effects. A formal test for whether the data should be combined (e.g., a Chow test), rejects the null hypothesis that a combined model is better.

Comment 3:
p. 15: "We include an array of individual and time-varying cluster-level characteristics, Xilcmt, …" How can this vector of variables be indexed to month and year of the individual's birth (since we have subscript mt) unless DHS surveys specifically ask questions for each individual corresponding to condition existing at their birth? X includes demographic variables like household size, age and sex of household head, etc. Since the Xs are indexed mt, this implies all these variables refer to the value of the Xs at a month and year the individual is born, i.e., household size, age/sex of household head, etc., when the girl/boy was born. This is unlikely. This needs clarification.

Thank you for catching this error. You are correct that the controls are not actually indexed to the month, but only the year an individual was born, and so the correct

indexing should be X_{ilct}. I have changed this throughout the text. Apologies for the confusion.

Comment 4:
p. 22: “The fixed effects and other covariates in our model control for location-specific and household-specific factors, time trends, and time-varying characteristics such as nightlights and temperature …” This statement gives the impression that the regressions control for household fixed effects. Isn't DHS data repeated cross-section? If so, you cannot control for household-fixed effects. Clarify or change the statement.

Thank you for the opportunity to clarify. You are correct that there are no household fixed effects, but only cluster-level fixed effects. I had meant to say that the fixed effects control for location-specific effects and time trends, while the other covariates control for individual, household and time-varying characteristics. I realize that the way the sentence was worded was confusing. Additionally, based on feedback from another reviewer to feature the assumptions more prominently, I have removed this paragraph from the limitations section on page 22 to the introduction. The relevant paragraph is reproduced below, and I believe it should be much clearer now.

> Since many factors may contribute to associations between conflict exposure and years of schooling, we use a regression framework that accounts for location-specific factors (e.g., certain areas being less likely to experience conflict and having a higher female educational attainment), cohort effects (e.g., global events), time-varying factors such as climate and rainfall, and household characteristics such as wealth. We thus compare children in the same location that experienced conflict during their primary school years, to children who did not --- a similar strategy to other recent work that examines the effect of conflict on child mortality (Wagner et al. 2018), maternal mortality (Kotsadam and Østby 2019), and immunizations (Østby et al. 2021). To interpret the estimates as causal, we rely on the assumption that there are no remaining unmeasured confounders that affect both conflict and educational outcomes, that vary within the same location over time (birth year). We believe this assumption to be plausible but acknowledge the limitations of inferring causal effects from observational data (see *Materials and Methods*).
